# Supplementary figures and images for: Experimental Granulomatous Pulmonary Nocardiosis in BALB/C Mice
Source: PLoS One. 2016 Jun 15;11(6):e0157475. doi: 10.1371/journal.pone.0157475 (PMC4909231; doi:10.1371/journal.pone.0157475)

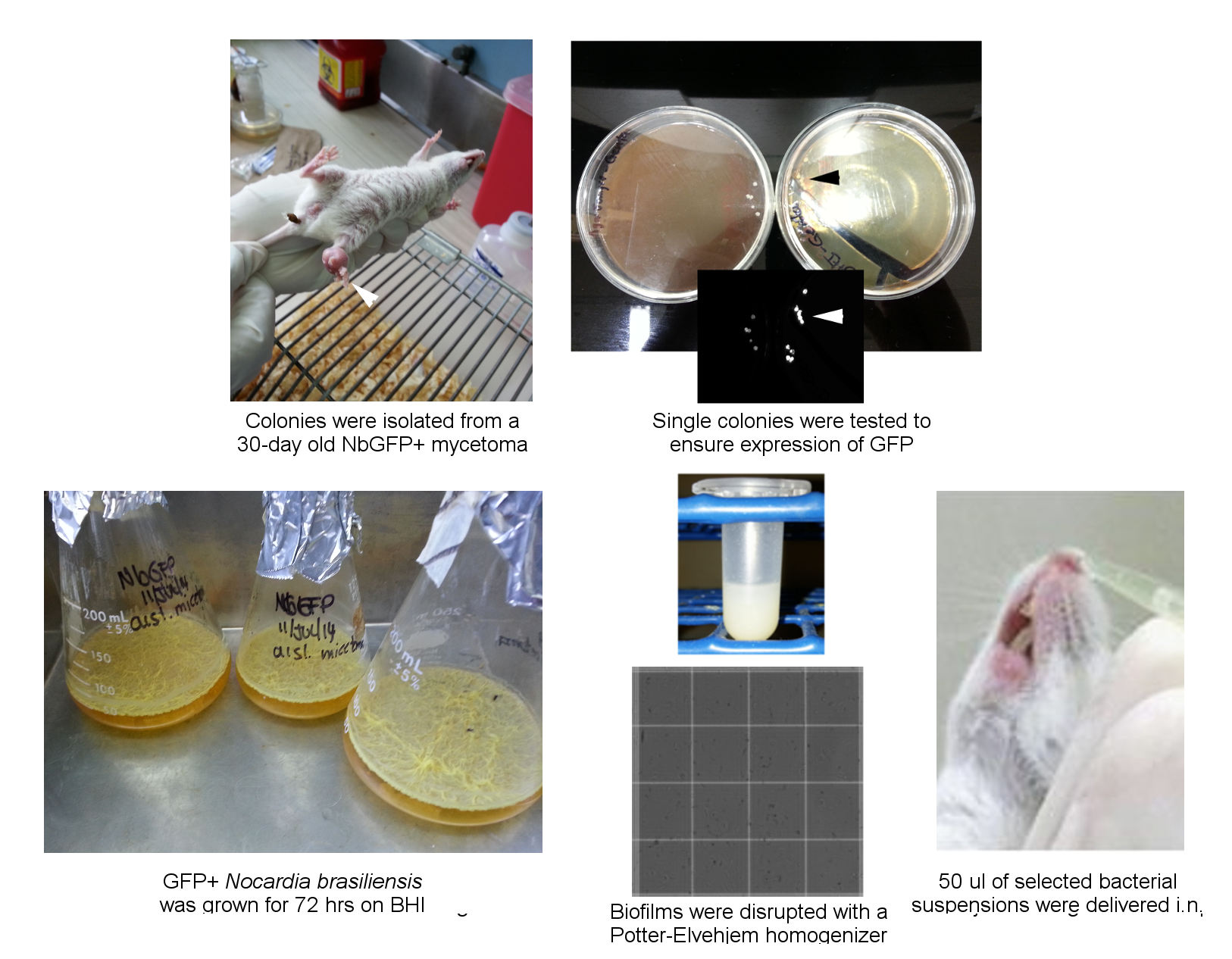

Supplement: S1 Fig — (TIF) [file pone.0157475.s001.tif]

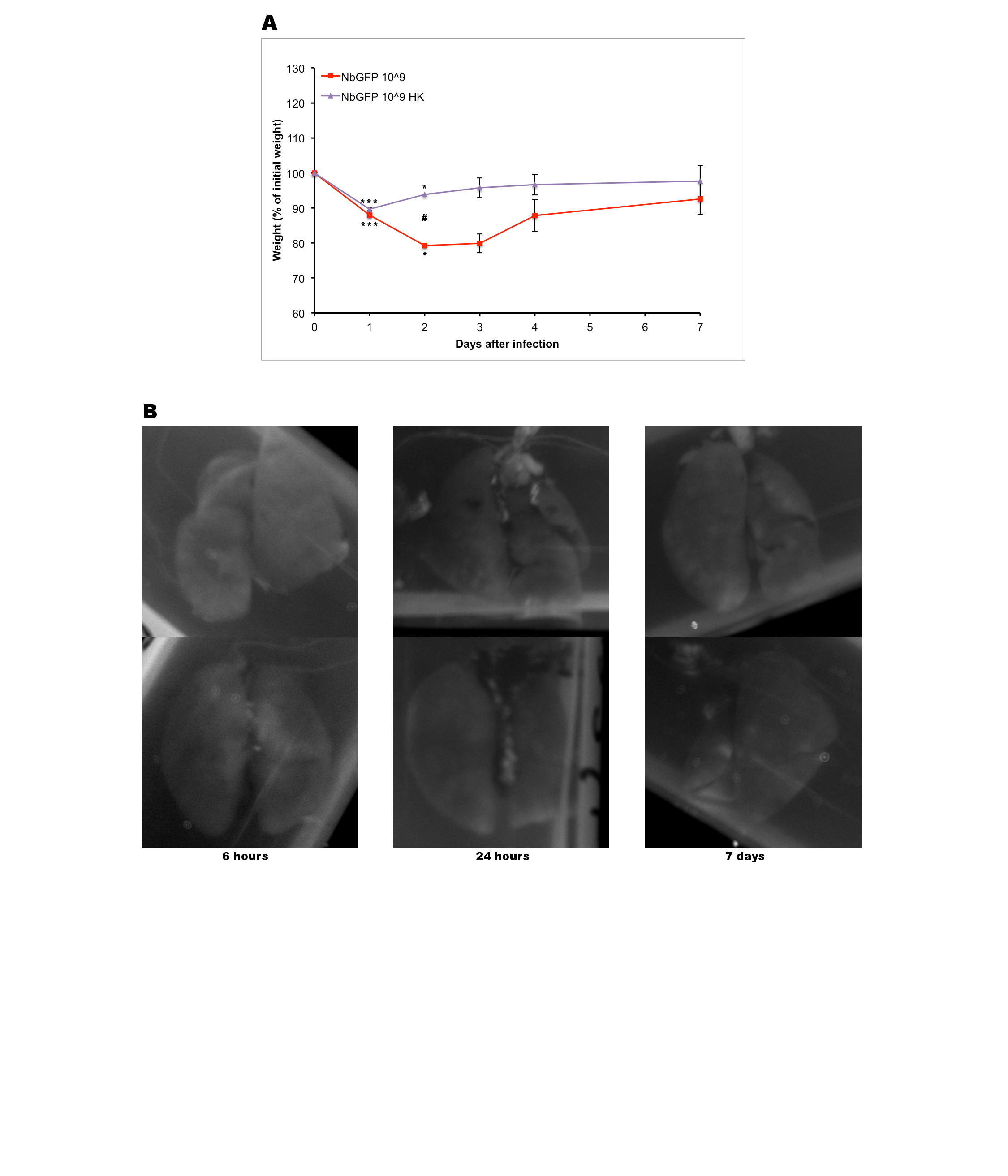

Supplement: S2 Fig — (TIF) [file pone.0157475.s002.tif]

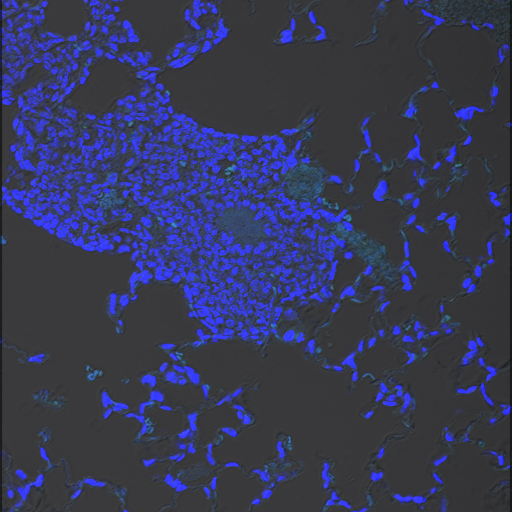

Supplement: S3 Fig — (TIF) [file pone.0157475.s003.tif]

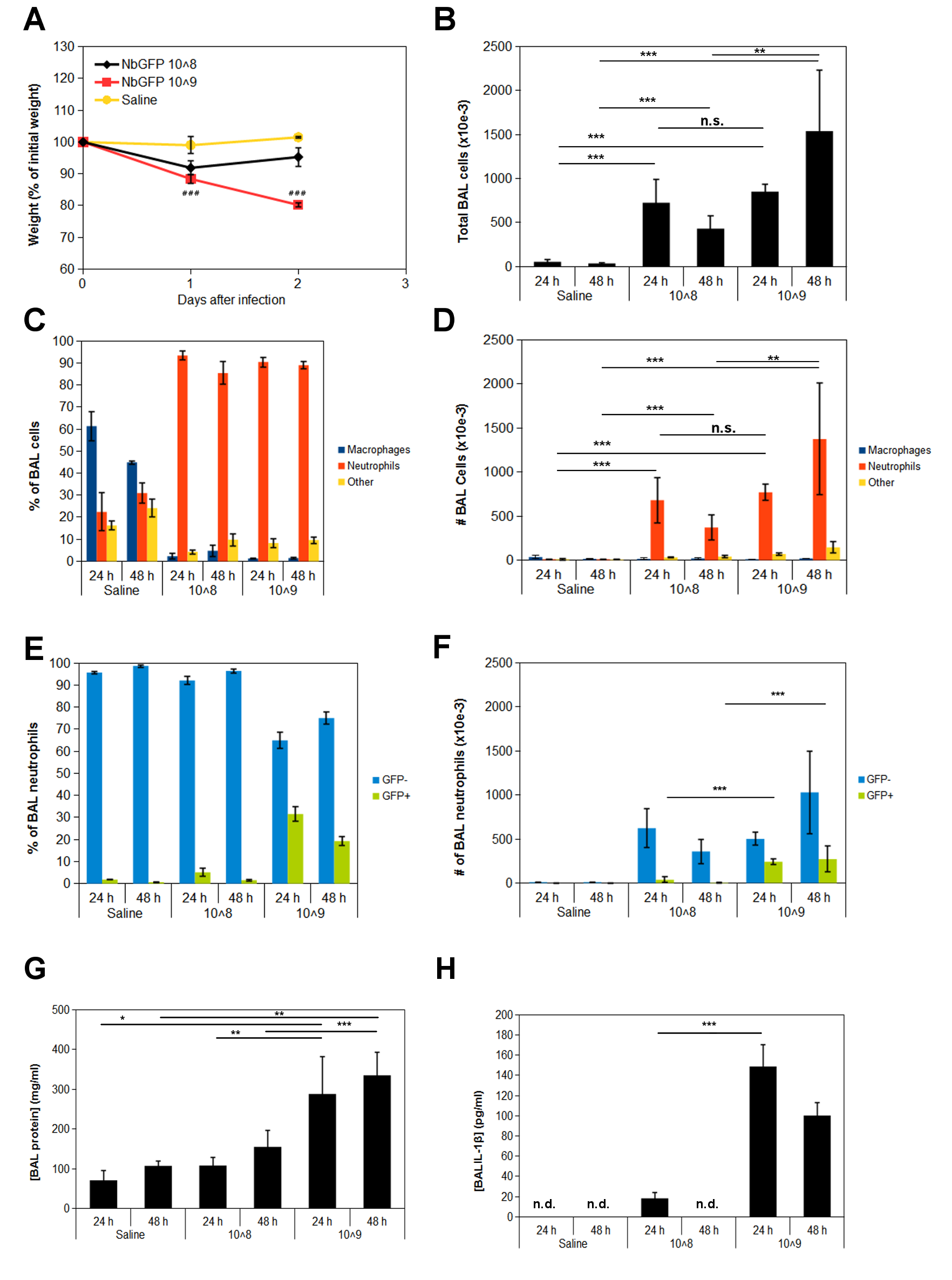

Supplement: S4 Fig — Graphs show average +/- s.e.m. (n = 5 per group) * p<0.05, ** p<0.01, *** p<0.001, ### p<0.001 108 vs. 109, n.d. = not detectable. (TIF) [file pone.0157475.s004.tif]
